# Hypertensive heart disease versus hypertrophic cardiomyopathy: multi-parametric CMR predictors beyond end-diastolic wall thickness ≥15mm

**DOI:** 10.1186/1532-429X-18-S1-P264

**Published:** 2016-01-27

**Authors:** Jonathan C Rodrigues, Stephen Rohan, Amardeep Ghosh Dastidar, Amy E Burchell, Laura E Ratcliffe, Emma C Hart, Julian F Paton, Mark Hamilton, Angus K Nightingale, Nathan E Manghat

**Affiliations:** 1 CMR Unit, NIHR Cardiovascular Biomedical Research Unit, Bristol Heart Institute, Bristol, United Kingdom; 2 Faculty of Medicine, University of Bristol, Bristol, United Kingdom; 3 Cardionomics Research Group, Bristol Heart Institute, Bristol, United Kingdom

## Background

Both American and European guidelines for the management of hypertrophic cardiomyopathy (HCM) advise that HCM be considered when LV end-diastolic wall thickness (EDWT) ≥15 mm in ≥1 myocardial segment. Hypertensive heart disease (HHD) is a common cause of left ventricular hypertrophy (LVH). Distinguishing between HHD and HCM is a frequent clinical conundrum. We aimed to identify predictors of HHD versus HCM with EDWT ≥15 mm, using multi-parametric cardiac magnetic resonance (CMR) including pixel-wise myocardial segmental EDWT analysis.

## Methods

HCM with EDWT ≥15 mm were identified from 2481 consecutive clinical CMRs between 2014-15. Those without a diagnosis of HCM (n=2428), with concomitant hypertension (n=21), apical HCM only (n=2) and severe renal impairment (n=1) were excluded. Analysis of 464 myocardial segments from 29 HCM subjects was performed.

HHD with EDWT ≥15 mm were identified from a separate prospectively maintained tertiary hypertension clinic database of 150 consecutive referrals. Hypertensive subjects with EDWT ≤15 mm (n=102), concomitant cardiac pathology (n= 17) and CMR contraindications (n=4) were excluded. Analysis of 432 segments from 27 HHD subjects were performed.

EDWT was measured by pixel-wise analysis. Segmental distribution of EDWT ≥15 mm, mean segmental thickness, segmental distribution of late gadolinium enhancement (LGE) were recorded. EDWT asymmetry, defined as >1.5-fold the opposing segment, was documented. The prevalence of other potential discriminators including systolic anterior motion of the mitral valve (SAM) and aorto-septal angulation (AoSA) were measured.

Unpaired T tests and multivariate logistic regression analysis was performed. Significance was set at two-tailed p < 0.05.

## Results

**Figure 1:**
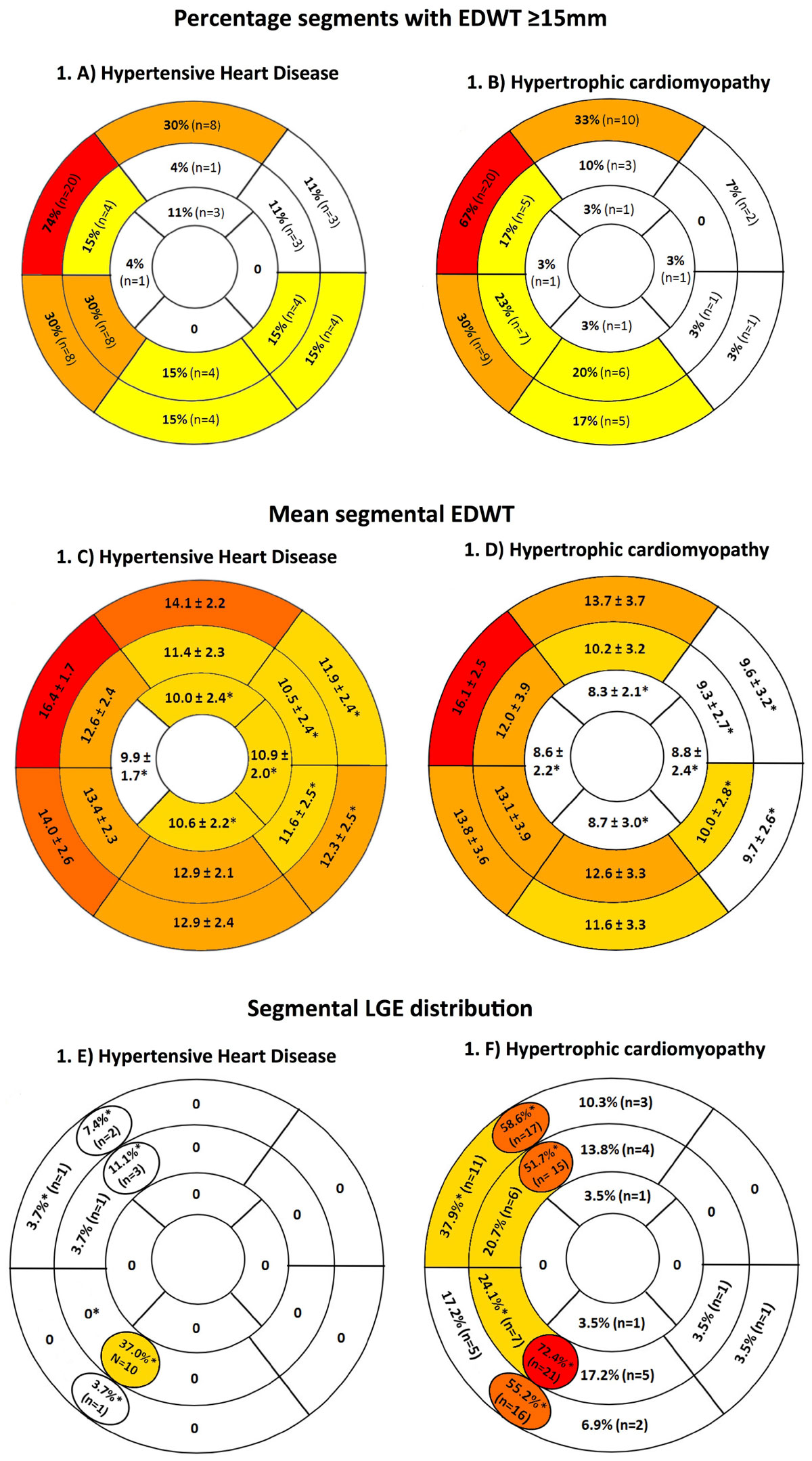
Figure 1

HHD and HCM cohorts were matched in age (HHD: 57 ± 13 vs HCM: 62 ± 10years, P=0.20) and gender (74 vs 59% male, P=0.28). HHD had significantly increased indexed LV mass (110 ± 27 vs 91 ± 31 g/m2, p < 0.05) and mass : volume ratio (1.44 ± 0.28 vs 1.29 ± 0.33, p < 0.05) compared to HCM but no significant difference in indexed LV volumes. There were no significant differences in the anatomical site or magnitude of maximal EDWT between HHD and HCM (Figure 1. A-D). The prevalence of LGE was significantly higher in HCM than HHD (Figure 1. E-F). Elevated indexed LVM, absence of SAM and absence of midwall LGE were significant predictors of HHD in the multivariate logistic regression model, but LV asymmetry was not (Table 1).

**Table 1:** Univariate and multivariate logistic regression analysis to determine predictors of hypertensive heart disease

	Univariate analysis		Multivariate analysis	
	OR (95% CI)	P value	OR (95% CI)	P value
Age (years)	0.99 (0.93 - 1.02)	= 0.20	...	...
Male gender	2.02 (0.65 - 6.27)	= 0.23	...	...
Indexed LVM (g/m2)	1.02 (1.00 - 1.04)	< 0.05*	1.05 (1.01 - 1.09)	< 0.05*
Mass : Volume ratio (g/ml)	5.03 (0.81 - 31.44)	= 0.08	...	...
EDWT Asymmetry	0.08 (0.02 - 0.33)	< 0.0001*	1.74 (0.11 - 28.96)	= 0.70
SAM	0.05 (0.01 - 0.21)	< 0.0001*	0.01 (0.00 - 0.25)	< 0.005*
Aortoseptal angle (degrees)	1.02 (0.97 - 1.08)	= 0.49	...	...
Midwall LGE	0.04 (0.01 - 0.15)	< 0.0001*	0.01 (0.00 - 0.18)	< 0.005*

OR = odds ratio, CI = confidence interval, LVM = left ventricular mass, EDWT = end-diastolic wall thickness, SAM = systolic anterior motion of the mitral valve, LGE = late gadolinium enhancement.

## Conclusions

There is a significant morphological overlap (location and magnitude of hypertrophy) between HHD and HCM. Both conventional EDWT ≥15 mm cut-off and LV asymmetry are poor discriminators. Elevated indexed LVM, absence of SAM and absence of midwall LGE are significant predictors of HHD. Tissue characterisation with LGE is unique to CMR and our findings support its extended use in cases of suspected HCM, particularly where there is concomitant hypertension.

